# Effect of core material draft ratio and denier on core spun yarn and denim fabric properties pre and post washing

**DOI:** 10.1016/j.heliyon.2024.e24965

**Published:** 2024-01-23

**Authors:** Md Anwar Hossain, Md. Amzad Hossain, Jabed Hossen Emon, Mohammad Tajul Islam

**Affiliations:** a Department of Textile Engineering, Uttara University, Dhaka, Bangladesh; b Department of Textile Engineering, Ahsanullah University of Science and Technology, Dhaka, Bangladesh

**Keywords:** Core-spun yarns, Draft, Spandex, Denim fabric, Denier

## Abstract

Core-spun yarn (CSY) is utilized for better fabric characteristics like stretchability, durability, and comfortability. The study aims to investigate the influence of spandex drafts of core-spun yarn on denim fabric characteristics before and after washing treatment. Two types of denim fabrics were produced from two types of core-spun yarn, namely 16 + 40D, and 16 + 70D by applying 2.8, 3.0, 3.20 spandex drafts for 16 + 40D, and 3.40, 3.50, 3.60 spandex drafts for 16 + 70D. Prepared denim fabrics were desized, and acid-washed and the properties of denim fabric before and after washing were investigated as a function of spandex drafts and deniers. Accurate count, twist, and better elongation percentage were observed at 2.80 draft for 16 + 40D CSY and 3.4 draft for 16 + 70D CSY, but a higher imperfection index (IPI) value was obtained on those drafts. The strength of the denim fabric prepared with 16 + 40D CSY and 16 + 70D CSY were higher at 2.8 and 3.6 drafts, respectively. Higher shrinkage (%), ends per inch (EPI), and fabric weight of denim fabric was obtained after washing compared to before washing. The width of both fabrics decreased when the fabric was washed. Exploring various drafts of core material and their correlations with yarn and fabric properties provides valuable insights for textile manufacturers seeking to produce denim fabrics with optimum quality.

## 1 Introduction

The application area of elastic CSY has widely increased in the textile industry due to the customers' demands for more pleasant, seasonable, and versatile clothes. This yarn can be made with spandex which comprises at least 85 % segmented polyurethane long-chain polymer to enhance elasticity and stretchability [[1], [2], [3]]. Generally, natural fibers are used as sheath covering and manmade or synthetic fibers are used as the core of the yarn structure [4]. The production of CSY aims to increase tensile strength, durability, comfort, aesthetics, dimensional stability, and other functional characteristics in the resulting yarn [5,6]. So, the fabric produced from this yarn has become more desirable for the customer than conventional fabrics and the use of these fabrics is rising precisely [7]. Several types of spinning systems can be applied to produce CSY such as rotor, ring, air-jet, and friction [8]. Elastic CSY is generally used to produce stretch fabric. So, this fabric has achieved leading characteristics of the staple fiber along with the advantages of elastic recovery and stretch [9].

Denim is one of the leading clothing products that are constructed with white weft yarn of coarser counts and 100 % cotton indigo-dyed warp and various weave designs of denim fabric are possible depending on the interlacement of these two sets of yarn [[10], [11], [12]]. Keeping the original size and shape due to body movement is one of the most significant characteristics of stretchable woven fabric. Denim fabric traditionally lacks stretchability and tends to be heavy. However, incorporating a core spun yarn with spandex in the center enhances the fabric's stretch capacity, providing added comfort [13]. So, stretch denim fabrics are produced from spandex blended yarn for special applications [14]. The weft yarn of denim fabric can be replaced with CSY, where cotton is used as the sheath and spandex is used as the core to improve the comfort and performance of denim [15]. The wearer feels pleasant because the sheath material cotton gives the required aesthetic performance and the core spandex enhances the stretchability and elastic properties [16].

Many researchers have studied the different characteristics of CSY like physical, mechanical, and elastic behavior, retraction [[17], [18], [19]], and the denim fabric properties such as mechanical and dimensional properties, elastic recovery and performance properties, tensile and tearing strength produced from CSY [[20], [21], [22]]. Choi et al. showed the influence of the spandex's feed-in angle and draw ratio on the structure and performance of CSY. They concluded that the higher the feed-in angle better the cover effect, and the highest tensile strength and elastic recovery were obtained at a draw ratio of 3.5 [23]. The impact of twist coefficient and spandex draft percentage on the bagging properties of elastic woven fabric were studied under tensile fatigue cyclic load. Baghaei et al. summarized that a lower twist coefficient and higher spandex draft reduce bagging deformation of woven fabrics [24].

In a study, Nilgün Özdil has compared different denim fabrics based on the bagging, stretch, and comfort properties with varying spandex ratios. He reported that the comfort properties of denim fabric had been enhanced with the increasing spandex content [25]. In another study, the mechanical properties of CSY were examined and optimal spandex draft percentages were found to dominate in determining the properties of yarn and increasing the production efficiency of the weaving process [26]. Qadir et al. applied different spandex drafts for spinning CSY with 40 and 70 deniers and observed that fabric properties (tear strength, stretchability, and recovery) increase with the increase of spandex draft. But the tensile strength showed the opposite trend [27].

Chemicals [[28], [29], [30]] and enzymatic finishing [31] are regularly applied to textile materials for various functional and aesthetic properties. Denim fabrics often undergo chemical and enzymatic washing processes. In this research work, the properties of CSY-containing denim fabric were examined before and after washing. To the best of our knowledge, washing effects on the properties of denim fabric prepared from CSY have not been reported in published literature. The properties of elastic CSY were measured for the different spandex drafts, and the effect of varying spandex rates and physical properties of woven cotton stretch denim fabrics (before and after washing) were also examined. It is found that the spandex rate significantly affects elastic CSY properties. In contrast, the spandex draft of CSY has no major impact on the manufacturing characteristics of final denim fabric properties.

## 2 Materials and methods

### 2.1 Materials

A roving of 100 % cotton fiber containing 70 % Cameroon-origin cotton and 30 % Mali-origin cotton and spandex filament with linear densities of 40 and 70 deniers was used. Details specifications of cotton fiber (for Cameroon-origin and Mali-origin) and spandex are mentioned in Table 1 and Table 2, respectively. Rucolase HCH (Rudolf, Bangladesh) and Asumerc NA (Asutex, Bangladesh) were used as desizing and wetting agents, respectively. Lava® con mex as a dispersing agent, Lava® sparse KTZ as an anti-back staining agent, and Lava® jeanspro-01 as a softener were supplied by DyStar, Bangladesh. Potassium permanganate, sodium meta bi-sulfite, and phosphoric acid were collected from Merck, Germany. Medium size (3–5 cm) fresh pumice stones (Turkey) were used for soaking.

**Table 1:** Specification of cotton fiber used in the preparation of core-spun yarn (CSY).

Properties	Cameroon-origin cotton fiber	Mali-origin cotton fiber
Spinning consistency index (SCI)	140	155
Micronaire value (μg/inch)	4.15	4.28
Strength (g/tex)	31.70	34.4
Upper half mean length (UHML) in mm	29.59	29.72
Uniformity index (%)	82.4	83.9
Elongation (%)	5.50	4.00

**Table 2:** Specification of spandex fiber used in the preparation of core-spun yarn (CSY).

Properties	40D/5F, Spandex yarn (Creora)	70D/5F, Spandex yarn (Creora)
Denier, d/9000 m, (dtex)	40.80	70.40
Tenacity (g/d)	1.38	1.13
Elongation at break (%)	454	594
Oil pick-up (%)	3.87	4.08
Package relaxation (%)	13.0	11.40
Running modulus (g)	11.3	12.40

### 2.2 Preparation of CSY

**Fig. 1:**
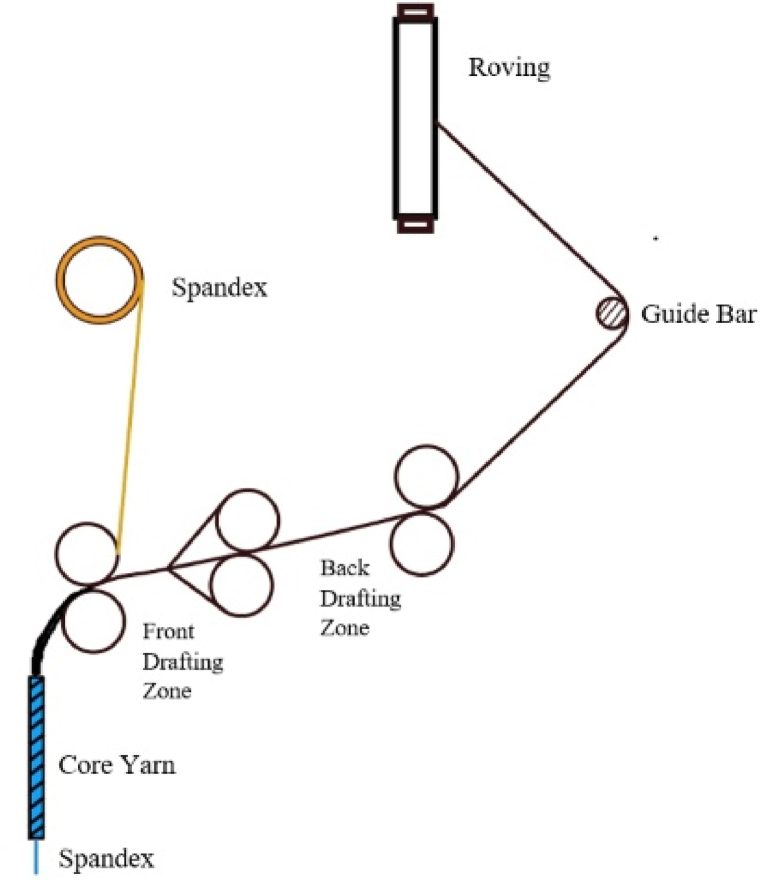
Arrangement for the spinning of core-spun yarn [32].

As shown in Fig. 1, a modified ring-spinning frame was used to make CSY. Spandex was inserted in the yarn's core through the spinning frame's front drafting zone. Two types of CSY, namely 16 + 40D and 16 + 70D, were made where the count of sheath yarn was 16s/1 Ne (carded) and the count of the core material was 40D and 70D, respectively. CSY was prepared using three different drafts such as 2.8, 3.0, and 3.20 for 16 + 40D CSY and 3.4, 3.5, and 3.6 for 16 + 70D CSY, respectively (Table 3). The range of drafts was chosen according to the industrial practices for the given deniers of the yarn i.e., 40 and 70.

**Table 3:** Process parameters used in the spinning of core-spun yarn (CSY).

Properties	16 + 40D CSY	16 + 70D CSY
Roving hank (Ne)	0.60	0.60
Spindle speed (rpm)	13000	13000
Ring diameter (mm)	42	42
Spacer size (mm)	3.5	3.5
Twist per inch	19.91	20.76

### 2.3 Preparation and washing of fabric

CSY was used in the weft direction to make the 3/1 Twill fabric with the fabric constructions mentioned in Table 4. After collecting the denim fabric from the loom, desizing was performed in an industrial horizontal sample washing machine (Yilmak, Turkey) with a material: liquor ratio (M: L) of 1: 10 using 2 g/L Rucolase HCH and 1 g/L Asumerc NA at 60 °C for 20 min. Desized fabric was then dried to make it suitable for an acid wash described by Elaissi et al. [33]. The pumice stones were soaked at room temperature for 10 min in a solution containing potassium permanganate (8 g/L) and phosphoric acid (1 mL/L) with an M: L of 1:2. After the desired soaking of pumice stone, dried desized denim fabric was treated with soak pumice stones at room temperature for 8 min using the same washing machine at 30 RPM. Pumice stones were unloaded from the machine after the acid wash. The acid-washed fabric was then neutralized with 5 g/L Sodium meta bi-sulfite, 2 g/L Lava® con mex, and 2 g/L Lava® sperse KTZ for 5 min to remove the stone dust and adhered chemicals. Finally, a softening treatment was performed using 3 g/L Lava® jeanspro-01.

**Table 4:** Construction used in the spinning of core-spun yarn (CSY).

Count	Spandex Draft%	Fabric Construction ($\frac{Warp\;count\times Weft\;count}{EPI\times PPI}\times Fabric\;width\;in\;inch$)
16 + 40D	2.80	$\frac{10X\;15.99}{83X59}X58.8$
	3.00	$\frac{10X\;16.02}{83X60}X58.7$
	3.20	$\frac{10X\;15.96}{84X59}X58.5$
16 + 70D	3.40	$\frac{10X\;16.05}{89X56}X60.5$
	3.50	$\frac{10X\;15.90}{89X57}X60.6$
	3.60	$\frac{10X\;15.85}{89X56}X60.5$

### 2.4 Characterization of CSY and denim fabric

#### 2.4.1 Measurement of yarn co-efficient of variation (CVm%), imperfection index (IPI), and elongation%

In this study, yarn CVm%, IPI, and Elongation% were evaluated by USTER® evenness tester-5, Switzerland, according to ASTM D1425/D1425M-14(2020) [34] under standard atmospheric conditions i.e., at 25 °C temperature and 65 % relative humidity. Standard atmospheric conditions were also maintained during all other testing mentioned below.

#### 2.4.2 Measurement of actual count and count strength product (CSP)

Auto wrap reel was used to produce a lea of 120 yards of yarn and this lea was then weighted by electric balance to calculate the actual yarn count. The strength of that lea was then determined using Lea Strength Tester, Statex, India, according to ASTM D 1578-93 [35]. Finally, CSP was determined by multiplying the count and strength of that yarn.

#### 2.4.3 Measurement of twist per inch (TPI)

A yarn twist tester, Testex, China was used to measure the level of twist of the yarn according to ASTM D1422/1423 [36] which can be equipped with auto stop and reverse for conventional or untwist/re-twist methods.

#### 2.4.4 Measurement of tensile strength of the fabric

Tensile strength determines the maximum force of textile fabrics until it ruptures. The grab test was applied according to EN ISO 1393-2 [37] to measure tensile strength using Titan, James Heal, UK. For experimentation, the test specimen was gripped in its center part by jaws of specified dimensions and extended at a constant rate until it ruptured. Then the maximum force was recorded.

#### 2.4.5 Measurement of tear strength of the fabric

Tear strength determines the fabric's resistance against tearing or the force required to propagate the tear once it is initiated. The tear strength test was conducted according to EN ISO 13937–1:2000 [38] using Elma Tear, James Heal, UK. The average reading was recorded as tear strength in N.

#### 2.4.6 Measurement of fabric ounces per square yard

GSM cutter, James Heal, UK was used for this test. The fabric was cut using the GSM (gram per square meter) cutter and the weight of the cut fabric was determined by electric balance. Then the ounces per square yard of denim fabric was determined according to ASTM D3776 [39].

#### 2.4.7 Measurement of ends per inch (EPI) and picks per inch (PPI)

The fabric was marked using a measuring scale at 1 inch to the warp and weft direction with a ball pen. Then the number of ends and picks within the marked point were counted using magnifying counting glass according to ASTM D3775 - 17e1 [40] test method.

## 3 Results and discussions

### 3.1 Effect of spandex draft on the properties of CSY

The properties of 16 + 40D CSY and 16 + 70D CSY are summarized in Table 5. Three different drafts of spandex were applied during the production of 16 + 40D CSY and 16 + 70D CSY. For spandex drafts 2.80 and 3.40 (the lowest among the three drafts) for making 16 + 40D and 16 + 70D, respectively, the actual count was very close to the nominal count. Moreover, better twist accuracy was also observed, but IPI and elongation% values were found higher. In the case of 16 + 40D, the CSP values decreased as the draft increased, but in the case of 16 + 70D, the opposite result was observed. In the case of 16 + 40D CSY, where 40D spandex is finer, the higher number of fibers per cross-section of yarn contributes to increased yarn strength than 16 + 70D CSY. On the other hand, for 16 + 70D CSY, the increase in spandex draft leads to finer spandex, allowing for more fibers to be incorporated into the yarn cross-section. This results in an increase in CSP as the draft is increased. The interplay among spandex denier, drafting process, and number of fibers per cross-section uniquely influences the CSP for each yarn configuration.

**Table 5:** Properties of core-spun yarn (CSY) for different draft ranges.

Count	Spandex draft (%)	Actual count	Twist/Inch	Elongation (%)	IPI	CSP	SD_csp_	CV_m_ (%)	SD_CV%_
16 + 40D	2.80	15.99	19.80	8.50	134	1840	3.95	12.85	1.02
	3.00	16.02	19.92	8.30	75	1800	4.20	12.77	1.11
	3.20	15.96	20.02	7.90	90	1790	4.02	12.71	0.99
16 + 70D	3.40	16.05	20.70	9.20	115	1700	4.32	13.20	1.16
	3.50	15.90	20.78	8.89	98	1730	4.65	12.95	1.05
	3.60	15.85	20.80	8.49	83	1749	4.88	12.50	0.89

### 3.2 Effect of spandex draft on the shrinkage (%) of denim fabric

**Fig. 2:**
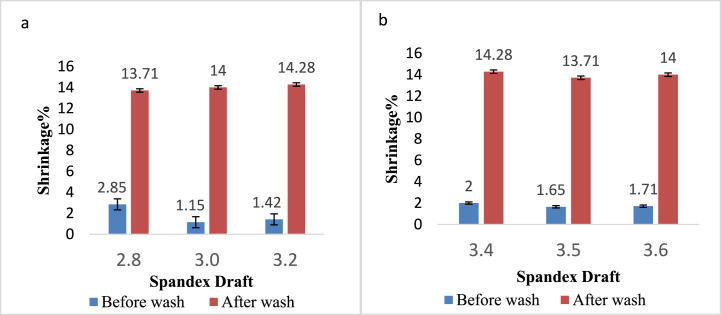
Shrinkage (%) of denim fabric before and after washing prepared from**:** a) 16 + 40D; b) 16 + 70D.

Fig. 2 indicates the impact of the spandex draft on the shrinkage% of denim fabric before and after washing. Both Fig. 2a and b represent that the shrinkage% of the washed sample was increased compared to the sample without washing. Kaynak et al. [7] have also made a similar observation. This can be attributed to the swelling characteristic of cotton fibers in water. The greater the amount of cotton fiber in the CYS, the higher the shrinking values. Spandex fibers also shrink greatly if hot water, steam, and hot air come into contact with a spandex content. Their elastic property made them shrink because of the rubber content [41]. But the spandex draft had no significant effect on the shrinkage of the denim fabric.

### 3.3 Effect of spandex draft on the width of denim fabric

**Fig. 3:**
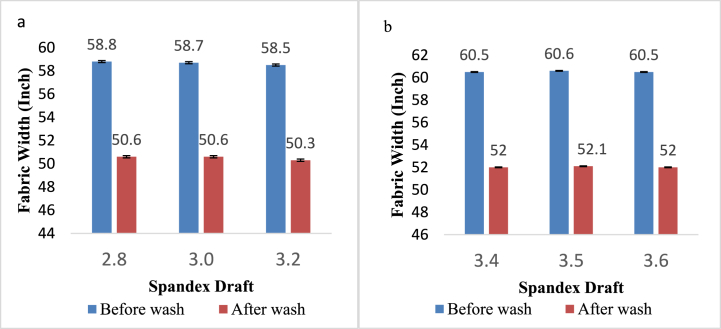
Width (inch) of denim fabric before and after washing prepared from: a) 16 + 40D; b) 16 + 70D.

Fig. 3a and b represent the effect of different spandex drafts on the width of denim fabric before and after wash. Fabric widths of washed samples were decreased compared to their before-wash state which was similar to previous studies by O.G. Ertaş et al. [12]. Since CSY was located along the width of the fabric, the shrinkage in the fabric occurred along the width after washing treatment due to the elastic property of CSY. The change in the spandex draft had no significant effect on the width of the CYS containing denim fabric.

### 3.4 Effect of spandex draft on EPI of denim fabric

**Fig. 4:**
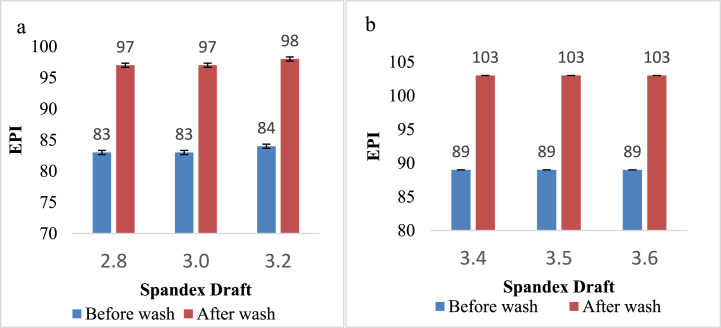
EPI of denim fabric before and after washing prepared from**:** a) 16 + 40D; b) 16 + 70D.

Fig. 4 illustrates the influence of spandex draft variation on the EPI of the sample before and after washing. Fig. 4a and b shows that the value of EPI was increased after washing the sample. Since CSY was located along the width of the fabric and the width of the washed sample was decreased due to the shrinkage, the number of warp yarn per inch increased in the washed sample. However, the change in the spandex draft had no significant effect on the number of EPI of the denim fabric.

### 3.5 Effect of spandex draft on the weight (Oz) of denim fabric

**Fig. 5:**
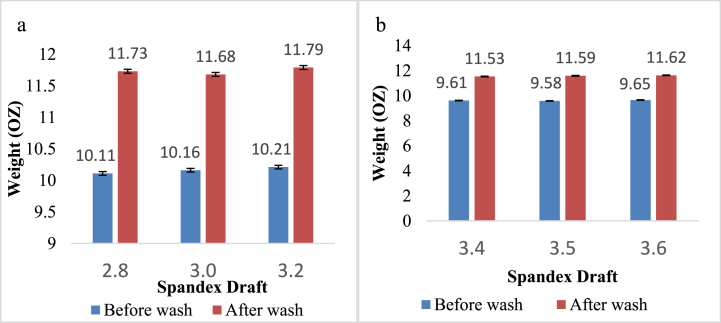
Weight of denim fabric (Oz) before and after washing prepared from**:** a) 16 + 40D; b) 16 + 70D.

Fig. 5a and b represent the variation of denim fabric weight (Oz) before and after washing for different spandex drafts. The sample's fabric weight (Oz) after washing was increased per unit length compared to before washing. It's worth mentioning that Kaynak et al. [7] also discovered an increase in fabric aerial density at post-laundering state, compared to before laundering. Since the fabric shrinkage occurred along the width and the number of ends per inch of the sample increased after washing, the weight of the sample also increased after washing. But the change in the spandex draft did not significantly affect the result. The weight of the fabric depends on both ends per inch (EPI) and picks per inch (PPI). Slight increase of weight may be attributed to the higher PPI of 16 + 40D.

### 3.6 Effect of spandex draft on tensile and tear strength of denim fabric

Table 6 shows the tensile and tear strength of stretched denim fabric after washing treatment was investigated. As only the weft yarn contained spandex, the result focused on the weft direction in both tensile and tear strength. The data showed higher tensile and tear strength for both 16 + 40D and 16 + 70D CSY in the case of low spandex draft which was also reported in M. B. Qadir et al. [15] studies.

**Table 6:** Tensile and tear strength of denim fabric after wash treatment.

Count	Spandex draft	Tensile strength			Tear strength		
		Mean (Newton)	SD	Elongation (%)	Mean (Newton)	SD	Elongation (%)
16 + 40D	2.80	356.41	2.08	66.14	28.02	1.79	42.00
	3.00	310.29	1.93	60.65	27.17	1.88	40.66
	3.20	317.57	2.01	72.28	26.36	1.79	39.66
16 + 70D	3.40	333.25	2.12	72.48	27.61	1.91	41.66
	3.50	327.51	1.86	71.43	25.89	1.90	38.66
	3.60	308.44	1.99	75.41	23.41	2.10	35.00

## 4 Conclusions

The performance and comfort characteristics of clothing are highly essential during use. Generally, efficient stretching of textiles is a welcome feature according to body posture. Due to stretchy features, spandex-containing denim fabrics have been more favored for occasional use in recent years. In this study, the impact of the spandex draft ratio and denier on the properties of denim fabric was examined. The desired result of CSY properties such as actual count, twisting precision, and elongation, was achieved at 2.80 and 3.4 drafts for 16 + 40D and 16 + 70D, respectively. Still, unfortunately, a higher IPI value was obtained that affected the quality considerably. The CSP value was higher at 2.8 and 3.6 drafts for 16 + 40D and 16 + 70D, respectively. Stretch denim fabric characteristics such as higher shrinkage (%), EPI, and fabric weight were noticed after washing as compared to before washing denim fabrics for both types of CSY. The findings also showed that the fabric width for both CSYs reduced when the fabric was washed. This research work will play a vital role in the optimized production of denim fabrics with enhanced stretchability, durability, and comfortability through the use of specific spandex drafts in core-spun yarns.

## Funding

This research has not received any fund.

## Data availability statement

Data included in article/supp. material/referenced in article.

## CRediT authorship contribution statement

**Md Anwar Hossain:** Writing – original draft, Supervision, Methodology, Conceptualization. **Md. Amzad Hossain:** Visualization, Investigation, Conceptualization. **Jabed Hossen Emon:** Visualization, Investigation. **Mohammad Tajul Islam:** Writing – review & editing, Supervision, Methodology.

## Declaration of competing interest

The authors declare that they have no known competing financial interests or personal relationships that could have appeared to influence the work reported in this paper.
